# Quantitative Systems Biology to decipher design principles of a dynamic cell cycle network: the “Maximum Allowable mammalian Trade–Off–Weight” (MAmTOW)

**DOI:** 10.1038/s41540-017-0028-x

**Published:** 2017-09-19

**Authors:** Matteo Barberis, Paul Verbruggen

**Affiliations:** Synthetic Systems Biology and Nuclear Organization, Swammerdam Institute for Life Sciences, University of Amsterdam, 1098 XH Amsterdam, The Netherlands

## Abstract

Network complexity is required to lend cellular processes flexibility to respond timely to a variety of dynamic signals, while simultaneously warranting robustness to protect cellular integrity against perturbations. The cell cycle serves as a paradigm for such processes; it maintains its frequency and temporal structure (although these may differ among cell types) under the former, but accelerates under the latter. Cell cycle molecules act together in time and in different cellular compartments to execute cell type-specific programs. Strikingly, the timing at which molecular switches occur is controlled by abundance and stoichiometry of multiple proteins within complexes. However, traditional methods that investigate one effector at a time are insufficient to understand how modulation of protein complex dynamics at cell cycle transitions shapes responsiveness, yet preserving robustness. To overcome this shortcoming, we propose a multidisciplinary approach to gain a systems-level understanding of quantitative cell cycle dynamics in mammalian cells from a new perspective. By suggesting advanced experimental technologies and dedicated modeling approaches, we present innovative strategies (i) to measure absolute protein concentration in vivo, and (ii) to determine how protein dosage, e.g., altered protein abundance, and spatial (de)regulation may affect timing and robustness of phase transitions. We describe a method that we name “Maximum Allowable mammalian Trade–Off–Weight” (MAmTOW), which may be realized to determine the upper limit of gene copy numbers in mammalian cells. These aspects, not covered by current systems biology approaches, are essential requirements to generate *precise* computational models and identify (sub)network-centered nodes underlying a plethora of pathological conditions.

## Introduction

Computational systems analysis can reveal hitherto unknown features of individual components of a biological process and, importantly, identify emerging properties underlying the process itself. While initial systems biology approaches were, often by necessity, reductionist and theoretical, they nowadays encompass entire molecular networks which increasingly rely on quantitative biological data. Molecular biology classically tends to be interpreted by phenomenological descriptions of biological processes, and subsequent analysis of their individual constituents. Therefore, an (r)evolution was needed directed towards the integration of biological data in computer models, which predictions may be not always straightforwardly interpretable through intuition.^1^

The realization that, amongst others, stochastic gene transcription may considerably impact on individual cell behavior^2^ has sparked a great interest in systemic approaches able to capture individual cell dynamics rather than representing the behavior of the average population. Experimental biology has thus shifted its focus from population-based qualitative analyses to single-cell-based quantitative analyses. This shift partially includes an emphasis on experimental methods such as microscopy techniques and flow cytometry, and the development of high throughput single-cell sequencing rather than biochemical techniques, such as Western blotting and Polymerase Chain Reaction (PCR), which are traditionally keyed to population analyses. Within this scenario, quantitative fluorescence time-lapse microscopy has helped greatly to elucidate many unknown protein properties which cannot be captured by in vitro, static analyses such as traditional biochemistry approaches. For example, the levels of the tumor suppressor p53, the “guardian of the genome”, have been shown to vary between cells and substantially oscillate depending on the cellular stress^3^, and its function to be compromised by incorrect cytoplasmic localization.^4^ Intriguingly, p53 oscillation amplitude and frequency depend on its subcellular localization, as well as association with other protein factors which exhibit an oscillatory behavior, such as circadian clock factors.^5^ Furthermore, the Nuclear transcription Factor kappaB (NF-ĸB)–which regulates expression of genes involved in inflammation and cell survival–shows robust nucleo/cytoplasmic oscillations upon stimulation by different doses of Tumor Necrosis Factor alpha (TNFα).^6^ Strikingly, these studies demonstrate that the frequency of spatial and temporal oscillations determines the nature of the resulting response and, in turn, depends on the amount and magnitude of upstream regulators.

The sheer size of the data generated by these methodologies, in which many individual cells may be followed not only statically but also in time, quickly becomes overwhelming. Thus, its integration into intelligible concepts often supersedes one’s intuition. To fully understand the data cohesion and analyze them to draw meaningful conclusions and to generate new hypotheses, it is crucial to integrate them into in silico mathematical models. These models have the ability to analyze molecular networks as a whole, precisely assigning the contribution of their components simultaneously. Such iteration between computation and experimentation, however, still requires the need to cleverly map a biological process under investigation with its underlying details, if the modeling outcome is indeed to be comprehensive. This approach is particularly relevant for those processes, such as the eukaryotic cell cycle, for which complexity is required to lend flexibility to respond timely to a variety of dynamic signals, while simultaneously warranting robustness to protect cellular integrity against perturbations.^7^

Here we propose how to integrate new and sophisticated experimental methodologies and definite computational frameworks to: 1) *Map* the mammalian cell cycle process, 2) *Measure* quantitatively and simultaneously the systems-level data that are required for the process to function dynamically, and 3) *Model* the process in silico. By a systemic exploration of quantitative properties (protein dosage) of cell cycle regulators, as well as their spatiotemporal dynamics (protein localization in time, therefore dosage distribution among cellular compartments), we will first provide a rationale for the relevance of these parameters for cell cycle timing, exemplified by the regulation of the Cyclin-dependent kinase (Cdk) inhibitor p27^Kip1^ (in the following indicated as p27), which controls timely phase transitions.^8^ Subsequently, we will review modern strategies to determine parameters experimentally, ultimately integrating them into computer models. We envision the creation of predictive, in silico models able to pinpoint how a change in the stoichiometry of molecular regulators, including protein complexes, and their spatiotemporal dynamics impacts on the timing of cell cycle transitions, thereby cell cycle robustness. These models may be employed to examine whether perturbations, e.g., reduction and increase in complexity, impact on both robustness and responsiveness of cell cycle dynamics.

Current computational models of cell cycle networks often rely on concentration thresholds arbitrarily chosen, which are required to switch from one cell cycle phase to another. Furthermore, they do not include protein localization or dosage variability. The overall strategy that we propose may explore systematically permissible protein ranges by an innovative, quantitative method that we name “Maximum Allowable mammalian Trade–Off–Weight” (MAmTOW), and predict the variations that may lead to loss of robustness. The evaluation of the extent to which removal of regulatory loops impinges on both robustness and responsiveness, constitutes a new way of exploring the effects that protein abundance, localization and complex formation have on cell integrity.

### Relevance of protein localization on cell cycle timing

Multi-level regulation of the cell cycle that includes response to stimulatory inputs, control of phase transition and adaptation to perturbations, requires cooperation of molecules interconnected in a dynamic network that warrants flexibility and robustness. These molecules act together in time and in different cellular compartments to execute the cell cycle program. While this program is unidirectional, multiple interactions and reactions occur simultaneously to mediate a timely succession of events. A property that still remains poorly understood is the cellular compartmentalization and, consequently, the change in localization of cell cycle molecules–in particular proteins–in time. We hypothesize that availability of proteins in the compartment where they excert their function modulates cell cycle timing; that is, correct protein localization is critical for cell cycle robustness.

**Fig. 1:**
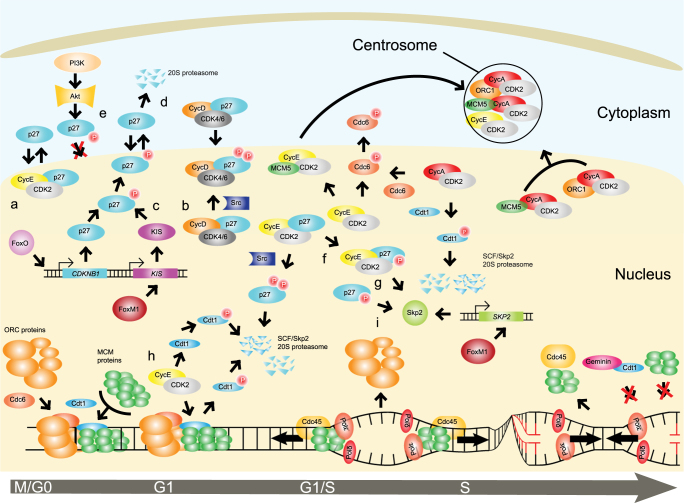
Schematic representation of processes occurring throughout cell cycle progression, exemplified by the role of the cyclin-dependent kinase inhibitor p27^Kip1^ (p27). **a** In quiescent cells, p27 accumulates and inactivates cyclin E/Cdk2 complexes–deputed to promote DNA replication at the G1/S transition-by sequestering them in a 1:1 stoichiometry and blocking their kinase activity (Supplementary References 1,2). **b** Mitogenic stimulation of quiescent cells leads to nuclear export of p27 mediated by cyclin D2 (Supplementary Reference 3) and the nuclear export protein CRM1 (not visualized) (Supplementary Reference 4). **c** Export of p27 is coincidental with its phosphorylation on Ser10 (Supplementary Reference 5) by KIS (Supplementary Reference 6) and possibly PKB/Akt (Supplementary Reference 7) kinases. **d** Upon cytoplasmic translocation, p27 is ubiquitylated and degraded through interaction with KPC ubiquitin ligase complexes (not visualized), reducing p27 protein content in both nuclear and cytoplasmic compartments through export and by degradation, respectively (Supplementary Reference 8). **e** Nuclear p27 is reduced further during the G0/G1 transition through its direct phosphorylation on Thr157 by cytoplasmic Akt kinase, preventing both de novo produced and exported p27 from (re)entering the nucleus (Supplementary References 9–11). Cytoplasmic translocation, degradation and p27 exclusion from the nucleus lead to a first wave of active cyclin/Cdk kinase complexes that allow entry of cells in G1 phase. During this phase, p27 regulation is altered: **f** activation of Cdk2 leads to phosphorylation of p27 on Thr187 and **g** recognition by the SCF(Skp2) ubiquitin ligase complex, which is followed by rapid degradation of p27 in the nucleus (Supplementary References 1,12). **h** This process leads to release of the inhibition of cyclin E/Cdk2 complexes and progression into S phase. **i** During S phase, Cdk2 continues to phosphorylate p27 on Thr187 and, in turn, SCF(Skp2) keeps p27 at low levels in the nucleus (Supplementary References 1,4,13–15)

The cell cycle is a prominent example of a biological process where many proteins follow a tightly regulated scheme over time (Fig. 1): synthesis and degradation, complex formation and dissociation, localization are parameters used to constrain cell cycle models. On the other hand, in these models protein localization is often approximated by a concerted decrease of protein availability in mathematical terms, akin to protein degradation. However, the consequences for the timing of cell cycle progression may differ dramatically between protein degradation and re-localization. For instance, the function of p53 may be compromised by its incorrect cytoplasmic localization, without any difference in net protein expression levels.^4, 9^ Similarly, untimely or aberrant cytoplasmic translocation of p27 may cause cell cycle disruption^10, 11^, in addition to relieving nuclear cyclin/Cdk kinase complexes from p27-mediated inhibition. In the cytoplasm, p27 interacts with non-Cdk proteins to ensure correct centrosome amplification and cytokinesis.^12, 13^ Even though levels of p27 are severely reduced after the G1/S transition, its localization re-wires the interactions it can establish in either nucleus or cytoplasm, effectively impacting on cell cycle temporal dynamics. Cyclin E/Cdk2 and cyclinA/Cdk2 complexes modulating G1 and S phases, respectively, are targets of p27-mediated inhibition and have a predominant localization in the nucleus. It has been reported that both cyclins E and A can also localize to the centrosomes.^14^ This localization proved to be important for a correct centrosome duplication and for initiation of DNA replication.^15, 16^ Canonical interactors of cyclins, Cdk2 and p27 have not been found at the centrosomes, suggesting that the interaction landscape of cyclins A and E differs depending on their cellular compartment.^17^ Strikingly, their inappropriate localization is detrimental in a number of cancers.^18^

These data imply that proteins may have functions outside their cognate compartment, and that computer models should appropriately include localization rather than emulating degradation simply by reducing to zero protein concentrations. As it becomes evident for the cyclins, native or spurious functions outside the canonical compartment may feedback on their availability inside the canonical compartment. Evidently, mislocalization impacts on protein availability at a definite timing during cell cycle progression, resulting in altered cellular dynamics and, ultimately, impacting on cell integrity. Importantly, *precise* dynamics of protein complexes, which are regulated by spatiotemporal localization and dosage of their components^19, 20^, shall be also considered. In conclusion, to comprehend how timely cell cycle transitions are achieved, accurate computational approaches shall include protein spatiotemporal dynamics.^21^ This may allow for the development of predictive models able to address the effect of protein mislocalization, e.g., changes in concentration and relative abundance, and its impact on the interaction landscape in the specific cellular compartments.

### Visualization of protein localization through time

Tracking protein localization has been possible since the introduction of the Green Fluorescent Protein (GFP) and its variants as tags to visualize proteins in single cells and in real-time by microscopy.^22^ Although nowadays tracking of fluorescent cell cycle protein derivatives is technically simple, it is challenging to do so while maintaining native protein expression levels and function, which rely on the chromosomal context of the encoding gene (i.e., introns, exons and proximity to (un)identified regulatory sequences). The conservation of these features requires the application of a more complex genetic engineering approach, by which a protein of interest can be visualized whilst ensuring that the spatial localization of its gene on the chromatin and its chromosomal context remain unaltered. The recently introduced CRISPR/Cas9 system allows editing genomes without altering the genetic context^23^, and offers a suitable platform for both gene-tagging and modulation of transcriptional regulation.^24–26^ In parallel, methods have been developed that separate the protein of interest from the fluorescent reporter, thus preserving its native conformation and function. Recently, a method has been developed that introduces a gene for a fluorescent protein within the same transcription unit as the gene of interest.^27^ A small sequence encoding a viral cleavage peptide was placed between the two genes, in order to separate the two encoded proteins post-translationally. The approach succeeded in retaining both native protein expression levels as well as providing a ratiometric readout to allow quantitative protein determination in single cells in vivo.^27^

A convenient way to visualize cell cycle progression is to utilize the variety of fluorescent reporters available to determine the *precise* timing of phase transitions. The Fluorescent Ubiquitination-based Cell Cycle Indicator (FUCCI) was developed based on the proteasomal turnover of fluorescently-tagged cell cycle proteins.^28^ By monitoring Cdt1-RFP and Geminin-GFP, which regulate alternatively activation (also called licensing) of replication origins to initiate DNA synthesis^29^, cell cycle dyamics can be monitored over time (Supplementary Fig. S1). Protein levels of Geminin and Cdt1 oscillate inversely: in G1 phase, Geminin is degraded and Cdt1 accumulates, thus promoting assembly of the pre-Replication Complex (pre-RC) at the replication origins (Cdt1-RFP is visualized as red fluorescence within the nuclei); in S/G2/M phases, Cdt1 is degraded and Geminin accumulates, thus inhibiting the formation of the pre-RC (Geminin-GFP is visualized as green fluorescence within the nuclei). Furthermore, the G0/G1 transition from quiescient to proliferating cells was monitored by using a fusion protein between the fluorescent protein mVenus and a Cdk-binding deficient p27 (p27K^−^) protein^30^, inhibitor of the cyclin/Cdk kinase activity and conserved in eukaryotes^31, 32^, in combination with the FUCCI system.

This method provides an effective way to easily separate cell cycle phases and measure the timing of phase transitions. However, it requires engineered cell lines while simultaneously reducing the usability of fluorescent tags in the chosen experimental setup. The cell cycle proteins used as markers for cell cycle transitions may not be completely inert, potentially changing the cycling behavior or the temporal phase distribution in the investigated cell type. Despite these potential challenges, the FUCCI system has become a leading system for cell cycle tracking in imaging experiments not only in cells but also in organisms^33^, and it has been used to screen the influence of anticancer drugs on cell cycle dynamics.^34^ Altogether, protein localization can be investigated by a combination of the FUCCI system, fluorescently-labeled proteins and fluorescence microscopy. Thus, the integration of modern genetic engineering and protein tracking technology enables spatiotemporal mapping of cell cycle protein dynamics.

### Altering spatiotemporal dynamics in the mammalian system

Experimental investigation of the impact that protein spatiotemporal dynamics may have on cellular processes and their molecular switches has been hampered by the continuous shortcomings of classic and modern techniques. For example, it remains difficult to dynamically alter protein localization, thereby protein dosage, on-demand. The use of proteins modified to contain in their sequence Nuclear Localization and Nuclear Export Signals (NLS and NES, respectively) to control localization are well known. However, they often lack temporal dynamics and regulation. Only recently strategies have been devised that enable the real-time activation of NLS and NES by simple light illumination. Several of such light-induced systems have been described^35, 36^ responding to different wavelengths of light. Many of these systems have some drawbacks, such as the requirement for external components to allow photoactivation, irreversible promoter activation by the light, or high background activation under non-induced conditions.

Recently, a more practical light-inducible system was introduced, based on optogenetic tools that enable controlling with light the nuclear import and export of tagged proteins in both budding yeast and mammalian cells.^37, 38^ Specifically, a Light-Inducible, fully reversible and genetically encoded Nuclear localization Signal (LINuS) was developed.^37^ This is based on a small tag, the Light-Oxygen-Voltage (LOV) domain, derived from the *Avena sativa* phototropin 1 protein (*As*LOV2), which can be fused to a NLS. Illumination by blue light triggers a conformational change of the AsLOV2 domain and the consequent exposure of the NLS, previously masked from the nuclear import machinery in the dark state, allowing it to be translocated selectively into the nucleus^37^ (Supplementary Fig. S2a). The NLS was subsequently replaced by a NES to enable nuclear export, a variant named Light-inducible nuclear EXport sYstem (LEXY)^38^ (Supplementary Fig. S2b). These systems were shown to function for transcription factor import^37^ into, and export^38^ out, of the nucleus. By mediating light-induced import of Cdk1, mitosis was induced in mammalian cells; this process requires a timely nuclear translocation of the cyclin B1/Cdk1 complex to the nucleus, which determines the commitment to mitosis.^39^ By demonstrating the applicability of the light-inducible system as proof of principle to investigate cell cycle control, this technique may be employed to a wide-range of cellular processes that require a timely activation upon translocation of pivotal proteins. Furthermore, it holds promises not only for on-demand, real-time control over protein localization, but also for single-cell localization studies (Supplementary Fig. S2c).

### Quantification of protein dosage

An argument in the (re)construction of multi-protein networks in silico is their ability to validate as well as predict the outcome of a multitude of biological experiments, especially when molecule concentrations are measured. In fact, these values serve as model parameters in computational models. For convenience, protein concentrations used in these models are often derived from biochemical determinations of average molecule numbers per cell.^40, 41^ These data are collected in PaxDB, a comprehensive Protein Abundance Database, which contains the publicly available experimental data of whole genome protein abundance averages across organisms and tissues, ranging from the budding yeast *Saccharomyces cerevisiae* to *Zea mays* to *Homo sapiens*.^42^ While the datasets of absolute protein concentrations are often incomplete, to date computational models have shown that multiple parameter settings may enable them to produce oscillating protein concentrations.^43^ This oscillatory behavior triggers cellular events, and is typically reproduced by the core machinery of eukaryotic cell cycle models, guaranteeing progression through the various phases after particular components, i.e., the mitotic cyclins, reach a definite threshold.^44^ However, if this threshold is not reached, a simulated cell cycle event does not occur.

In order to generate accurate models and compute their robustness, it is desired to elaborate further on the experimental technologies currently available, which should be tailored to *precisely* quantify protein concentrations as well as cell-to-cell and intracellular spatiotemporal variability. Although absolute protein concentrations are difficult to measure experimentally, a new technique, named Protein Quantitation Ratioing (PQR), has been recently developed to measure the stoichiometric ratio between a GFP (or potentially any other fluorescent) reporter and a protein of interest during protein translation in single cells in vivo^27^ (Supplementary Fig. S3). As a result, the fluorescence intensity is proportional to the molecule number of the protein in the cell. Experimental and in silico evidence on individual signaling pathways suggests that the fold change of protein dosage rather than absolute protein levels may determine a cellular response.^45, 46^ Kitano and colleagues have developed further on this line, addressing protein dosage elegantly by determining upper^47, 48^ and lower^49^ expression limits of cell cycle proteins in budding yeast and in the fission yeast *Schizosaccharomyces pombe*.^50^ Strikingly, their findings indicated that neither the fold change nor the absolute concentration increase of the investigated proteins were leading for a protein’s upper expression limit, but rather their stoichiometric balance in dimeric protein complexes. Thus, we envision that an imbalanced protein dosage may disturb the stoichiometry within multiprotein complexes that are responsible for the passage through phase transitions. This imbalance ultimately would destroy the network robustness, resulting in a perturbed cell cycling, thereby in a reduced cellular viability.^51, 52^ This would also occur because changes in the stoichiometry within multiprotein complexes does result not only from changes in protein dosage, but also from an altered protein localization.

Availability of the empirically determined absolute concentration of a protein enables estimation of the relative expression of its partner within a multimeric complex. However, this information may be not obtained when only the variability of a protein through time–derived from fluorescently tagged-protein dynamics by fluorescence microscope – is known. That is, absolute spatiotemporal concentration data measured for a large panel of proteins in a similar system shall also yield information about protein dosage. Hitherto, the most efficient system to measure protein concentrations in single living cells is offered by the Fluorescence Correlation Spectroscopy (FCS) technique, which enables determination of the absolute concentration of fluorescent (bio)molecules by measuring fluctuation of the fluorescent intensity of a molecule in a given and fixed volume^53, 54^ (Supplementary Fig. S4a). Several FCS-related techniques exist that may be suitable to measure protein concentrations in cellular compartments, migration speeds over membraneous structures, and quantify protein complex formation. Through a pinhole only a small volume of the sample, called confocal volume, is monitored. Fluorescent particles fluoresce whenever they pass through this volume. By sampling the fluctuation intensity as fluorescent particles travel in and out of the confocal volume at fixed time intervals, an autocorrelation plot is generated (Supplementary Fig. S4a). This plot yields *precise* information about the average number of fluorescent particles and average diffusion times, ultimately determining protein concentrations. For optimal sensitivity FCS requires molecule concentrations to be in the nanomolar to picomolar range, which makes this technique ideally suited for fluorescent proteins in living cells.

An extension of the FCS technique is called Fluorescence Cross-Correlation Spectroscopy (FCCS), and it is employed to measure the diffusion of multiple fluorescent molecules simultaneously (Supplementary Fig. S4b). Measurements of intensity fluctuations in the confocal volume of each fluorescent molecule allow for the determination of complex formation and of the fraction(s) of complexed and non-complexed molecules. Thus, by selectively targeting cellular compartments, such as nucleus or cytoplasm, FCS measurements combined with conventional confocal microscopy imaging provide information about complex formation between two selected fluorescent molecules, also measuring their spatiotemporal dynamics. Interestingly, recent developments allow for a full automation of FCS/FCCS in living cells with simultaneous confocal imaging, high-throughput characterization of a large number of proteins, and time-resolved characterization of protein complex dynamics throughout an entire cell cycle.^55^

In summary, FCS and its variants require great care in instrumental setup and calibration^54^, but they offer great promises to determine biological variations in time and space of individual proteins and complex formation. These data may then be converted to kinetic parameters, which represent an input for detailed computational models.

## Engineering mammalian cells to *precisely* determine protein dosage

As outlined earlier, it may be hypothesized that alteration of the protein dosage impinges on both cell cycle timing and robustness. Cell cycle robustness may be compromised by out-of-boundaries protein expression, and its violation may be not identical quantitatively among various proteins and cell types. As a consequence, we propose that a compromised robustness due to an altered expression of a cell cycle protein may be balanced by simultaneously altering the expression of one of its partner within a protein complex. This may be particular relevant for those complexes that are responsible for biochemical switches at cell cycle transitions. Considering the likelihood of this scenario, the importance to quantify the expression limit of individual cell cycle proteins, and to determine how its changes affect cell cycle timing and robustness, becomes evident. The *precise* stoichiometry of proteins within complexes can be integrated together in new and/or existing in silico models of cell cycle control, which would then be able to examine how gene dosage–through their effects on multiprotein complex stoichiometry–may impact on both robustness and responsiveness of cell cycle dynamics.

**Fig. 2:**
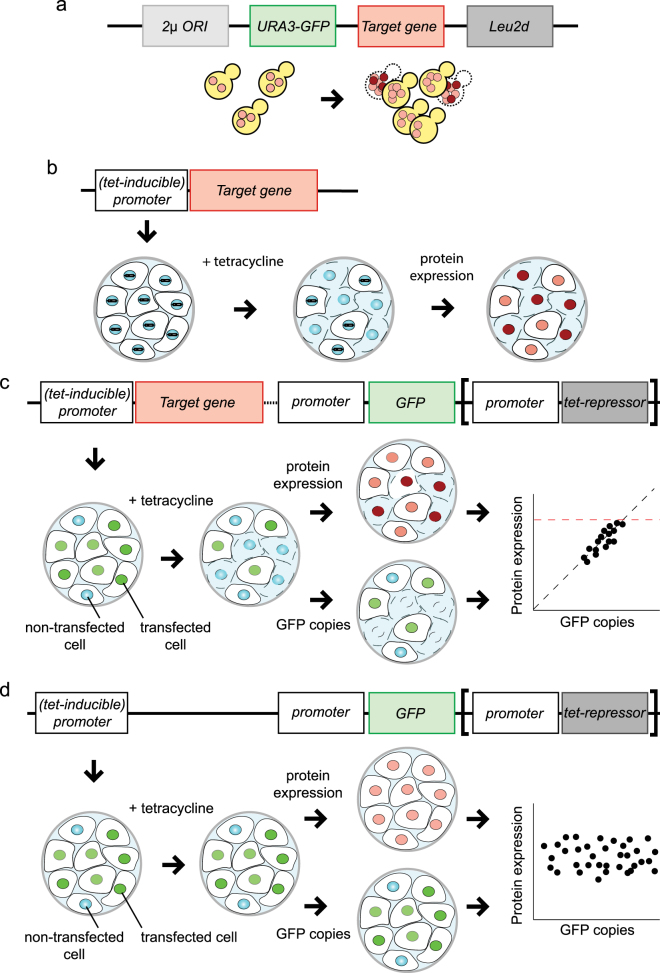
Cloning strategies to explore the upper limit of protein expression in mammalian cells by transient transfection methods. **a** The transient system as proposed by Kitano and colleagues in the budding yeast *Saccharomyces cerevisiae* (see text for details). A GFP reporter is used as a quantitative readout of relative plasmid copy number, and it can be used to determine the upper limits of target gene expression. When the upper limit is reached, yeast cells will not be viable and a cut-off in fluorescent signal is measured. **b** The simplest example of a transient system for mammalian cells that may be used to determine upper protein expression limits. A plasmid bearing a target gene (visualized in red) driven by its native promoter is transfected into a cell line. The expression level may be boosted by using a switchable tetracycline-inducible (tet-inducible) promoter, which requires a cell line expressing the tet-repressor. By varying the amount of transfected plasmid, expression level of the target gene may be regulated. Similarly to the system shown in **a**, cells that contain a too high number of plasmid copies–thus, protein amounts beyond the viable expression limit–will not survive. Relative protein expression upper limits may be determined by immunostaining, and comparison with non-transfected cells. **c** Elaborated version of the approach in **b**, where quantification is achieved by a fluorescent reporter (e.g., GFP). A cassette containing the tet-repressor may be inserted to allow for an inducible protein expression by using a tet-inducible promoter. In cells expressing the tet-repressor, or when another promoter is chosen, this cassette is dispensable. The signal obtained from the fluorescent reporter shall be proportional to the target gene expression, and may be used as a readout for relative plasmid copy number, thereby protein expression. This system is independent from antibody staining. **d** Control experiment of **c**, where the target gene is lacking. In this construct, the GFP signal should show no correlation with the target protein expression. The upper limit of plasmid copies may also be determined by the allowed upper level of GFP expression that preserves cell viability, thus setting the range for measurements

To determine the impact of protein dosage and localization on cell cycle dynamics is challenging, due to the limited experimental toolset available that allows for a quantitative, incremental variability of these parameters in vivo. In fact, systematic control of protein abundance in mammalian cells is limited by the currently available experimental techniques to artificially and dynamically modulate the amount of gene copy numbers. Conversely, in both budding and fission yeasts, a dedicated system named genetic Tug–Of–War (gTOW) has been developed to determine the maximum dosage of proteins that a cell is allowed to express yet being viable.^47, 48, 50^ In this approach, a transcription unit of choice is cloned into a yeast expression plasmid, and an artificial increase of the plasmid copy number per cell is achieved by a selection marker. Practically, a leucine-deficient yeast strain is transformed with a plasmid carrying a gene of interest and a gene relieving the leucine-deficiency (*leu2d*). The capacity of the cells to grow on a leucine-deficient medium is linearly proportional to the expression of the *leu2d* gene, giving yeast bearing many plasmids (thereby, many gene copies) a growth advantage. Expression of the gene of interest, however, may limit the maximum plasmid copy number when the gene product is toxic upon its overexpression (Fig. 2a). Hence, growing the yeast strain in a leucine-deficient medium will lead to a gTOW game where the two genes are at opposite ends of the rope. This enables a *precise* determination of the highest copy number of a given gene at which a cell still thrives. Strikingly, the ceiling of expression differs markedly among genes. The yeast gTOW system cannot be adapted to the mammalian system due to the limitation represented by the selection marker to increase the plasmid copy number.

In this section, by elaborating on the yeast gTOW system, we explore the possibility to perform experiments yielding to comparable data in mammalian cells. Through the availability of modern techniques, such as the CRISPR/Cas9 technology, we propose how the limit of protein dosage in mammalian cells may be determined, and modulated, quantitatively. We envision the development of strategies to create cell lines carrying a synthetic cassette, which allows for a measurable and tunable expression of a protein of interest.

### Measuring the upper limits of protein expression in mammalian cells

One of the key advantages of the transient gTOW system is that it does not require the generation of specifically engineered yeast strains for every protein that is to be investigated. Quite on the contrary, the system relies on the runaway replication of episomes independent of the host chromosome. For fast screening of a vast amount of proteins, a transient system presents a versatile and cost-effective option. Transfection of mammalian cell lines with a transient, plasmid-based, system carrying an expression cassette consisting of a gene of interest (for our scope, a cell cycle gene would be the target gene) expressed under the control of a promoter of choice, offers an equally fast and versatile system depending on the desired level of expression. Variability in expression is controlled through the amount of plasmids transfected and the promoter that is selected for expression. For example, a switchable tetracycline-inducible promoter may be used to control the (over)expression (Fig. 2b). The cassette contains a *GFP* reporter gene controlled by a constitutively active promoter, preferably yielding low, but measurable protein expression. In an experimental setup, the critical parameters that determine protein expression ranges (promoter strength, cell line transfection regime, optimum dosage of doxycycline/tetracycline) are optimized. If a too high-potentially lethal-amount of the gene product may be expressed, cells may undergo apoptosis, whereas cells expressing a tolerable gene copy number, thereby protein amounts, are viable and analyzed (Fig. 2c). GFP serves as a readout for the amount of transfected DNA. Specifically, a linear correlation is expected between GFP copies and the levels of a target protein, whereas no correlation is expected in a construct lacking the target gene but carrying GFP under a constitutive (non-induced) promoter (Fig. 2d).

As indicated, tetracycline inducibility allows for an exact determination of the experimental startpoint. After optimizing the appropriate experimental parameters, the maximum expression limit can be determined by measuring the maximum GFP levels, hence the maximum relative plasmid copy number, by fluorescence microscopy and/or flow cytometry. These results may be compared to immunofluorescent staining of the protein of interest to validate the linearity between GFP signal and protein dosage (Fig. 2c). However, whereas immunofluorescence is capable of protein dosage quantification^41^, it cannot be used easily to compare relative plasmid presence among different proteins of interest. Therefore, measurements of GFP intensity would be preferred as, after calibration, these allow to compare protein dosage between samples (as they should reflect the relative plasmid copy number akin to the determination of plasmid copy number in yeast). By comparing the fluorescence levels of induced samples and non-induced controls, the cut-off relative plasmid copy number, hence the expression range of the protein of interest allowed by the cell, may be determined. The advantages of this method are multifold: (i) it is relatively fast and fully transferable between cell lines, thus enabling the comparison of different cell types, (ii) it relies exclusively on transient transfection, and (iii) it allows for an easy co-expression of multiple cell cycle proteins at the same time and in the same experiment. After optimizing the experimental parameters, the data derived are expected to be similar to those obtained from gTOW experiments.

A non-native tetracycline expression unit and its variants have been successfully employed in recent studies to control gene expression in mammalian cells, which also carry an inducible target gene coupled to a reporter^56, 57^ or that additionally respond to a light-switchable transactivator.^58^ Interestingly, the tetracycline-inducible system has been also utilized to control cell cycle fate, specifically to suppress malignant growth and induce apoptosis in cancer cells.^59^

### MAmTOW: a CRISPR/Cas9-based gene expression system

A complementary strategy to measure gene dosage may be envisioned taking advantage of the CRISPR/Cas9 technology^23^. It has enabled not only directed knockout of genes in cells of various backgrounds, but also gene or gene-promoter-replacements. Contrarily to techniques that rely on random integration, the CRISPR/Cas9 technology allows for a high degree of control by allowing site-directed modification of genomes. Thus, it is expected to yield more reliable data, due to the fact that artefacts due to spurious and/or gene-disruptive integrations shall be more controllable as compared to other genetic engineering strategies. As gene placement may have a substantial impact on transcription dynamics, it offers advantages such as retroviral transduction or transfection. By making use of CRISPR/Cas9, here we conceive an innovative molecular methodology, which we coin as “Maximum Allowable mammalian Trade–Off–Weight” (MAmTOW). This methodology uniquely describes the functioning of a synthetic cassette integrated into the genome of mammalian cell lines, which replaces the endogeneous promoter of a target gene to allow for its tunable expression and quantification (once again, for our scope, a cell cycle gene would be the target gene). Integration of gene cassettes is less versatile as compared to a transient transfection; however, the extra burden is a trade-off for quality, as CRISPR/Cas9-engineered cell lines are expected to exhibit a native-like behavior as compared to cells transiently transfected with plasmid DNA.

**Fig. 3:**
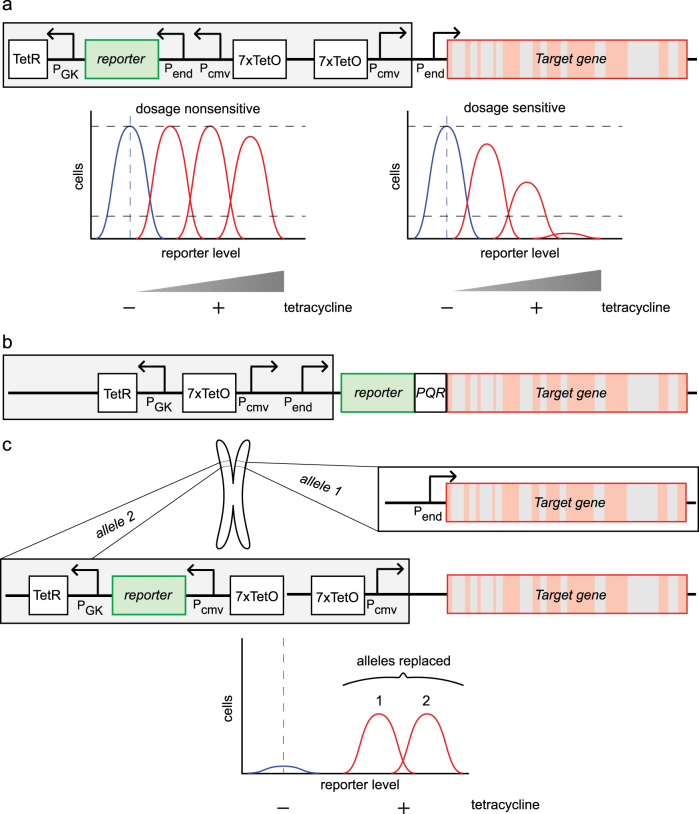
Maximum Allowable mammalian Trade–Off–Weight (MAmTOW) methodology to explore the upper limit of protein expression in mammalian cells by stable integration via CRISPR/Cas9. **a** Through CRISPR/Cas9 the gene cassette (gray background) is integrated in front of the target gene on both gene’s alleles. The cassette consists of (from left to right): a tet-repressor (TetR) under the control of the P_GK_ promoter; a fluorescent reporter gene (e.g., GFP) controlled by the endogenous promoter of the target gene (P_end_); a cytomegalovirus promoter (P_CMV_) controlled by a Tet-operator unit (TetO), which is repeated seven times; and an identical Tet-operator unit in the sense-direction controlling a P_CMV_ promoter. The target gene (visualized in red, with introns indicated in gray) is intact and in its native context. Under non-induced conditions, both reporter and target genes are transcribed according to the target gene’s native regime, while TetR is constitutively transcribed. When tetracycline is added, TetR units are removed from the Tet-operator activating P_CMV_, and a proportional boost of transcription of both reporter and target genes occurs. By determining cell viability in the presence of increasing tetracycline levels, the upper limit of protein expression relative to non-induced conditions may be determined. **b** In an alternative scenario, the target gene is modified on both gene’s alleles to be fused in frame with the fluorescent reporter gene and a protein quantitation ratioing (PQR) domain upstream. The translated polyprotein is cleaved by the PQR domain, resulting in a stoichiometric ratio between reporter and target proteins. **c** In addition to a double-allele replacement assumed in **a** and **b**, also single-allele replacements may occur. These can be identified by the level of fluorescence associated to the reporter protein. In case the strategies in **a** and **b** hinders native gene transcription of the target gene under non-induced conditions, cell lines may be selected that bear only single-allele integrations (assuming a diploid target cell line). In the gene cassette used for this strategy, one allele is intact, while the other one carries the cassette shown in **a** (gray background) upstream of the target gene. The placement is such that the modified allele is completely controlled by tetracycline induction

A replacement of the two alleles of the reporter (cell cycle) gene through CRISPR/Cas9 is shown in Fig. 3a. The entire cassette may consist of: (i) a tetracyclin repressor (TetR unit), encoding a bacterial repressor of the *TET* resistance gene, controlled by the weak mouse phosphoglycerate kinase 1 promoter (P_GK_), (ii) a reporter gene unit, encoding GFP or another suitable fluorescent protein, controlled by both the native, endogenous promoter (P_end_) of the gene of interest and a tetracycline-inducible promoter, consisting of (iii) a strong cytomegalovirus promoter (P_CMV_) preceding several copies–typically six or seven^56, 58^ –of a Tet-operator sequence (TetO). The TetR unit is continuously produced by placing a constituvely active promoter (P_GK_) to the 3′-end of the fluorescent reporter gene, to prevent target gene transcription under non-inducing conditions (Fig. 3a). The cassette may be followed by a transcription unit constituted by the endogenous target gene bearing its native promoter P_end_. To prevent polymerase read-through transcription, the fluorescent reporter cassette and the endogenous target gene cassette should be placed in reverse orientation relative to each other.

Both fluorescent reporter and target genes are regulated by identical control elements. To ensure a reliable and robust, non-leaky induction of both genes, the number of TetO copies may need to be optimized so that, in absence of tetracycline, P_CMV_ is repressed but both the target gene and the fluorescent reporter gene are transcribed normally. Upon addition of tetracycline (or the more stable doxocycline analog), (over)expression may be induced for both target and reporter genes, and a linear correlation between these should be verified by either fluorescence microscopy or flow cytometry (Fig. 3a). By dose-response analysis, the upper expression limit of any target gene may be determined in presence of tetracycline. As with the gTOW methodology and the transient (over)expression of genes described above, the upper level of gene expression may be measured and serves as a readout for the expression ceiling of a target gene with relation to cell viability. In fact, the maximally allowable dosage of a definite cell cycle regulator may impact on the proliferative capacity, thereby on the cellular robustness. It is conceivable that, in a non-induced scenario, transcription from the native promoter of some target gene might be quenched by the TetR elements. If this would occur, cells with single-allele replacements may be selected to ensure presence of the protein of interest (albeit at a lower level) while retaining inducibility.

A methodology recently developed, the Protein Quantitation Ratioing, may be employed to determine quantitatively a stoichiometric ratio between the fluorescent protein reporter and the target protein in vivo.^27^ Insertion of a tag, a Protein Quantitation Reporter (PQR), between the fluorescent reporter gene and the target gene, allows for co-transcription and co-translation of both genes, obviating the need for a separate transcription module for the reporter gene (Fig. 3b). After translation the inserted PQR sequence leads to its own removal by interfering with polypeptide elongation, effectively leading to its excision and separation from the fluorescent reporter and the target protein. Since the PQR sequence serves as a “yoke”, both fluorescent reporter and target protein are now present in an equimolar ratio. Therefore, the intensity of the fluorescence reporter is directly proportional to, and a measure of, the amount of the target protein.

As the CMV promoter (P_CMV_) and the TetO cassette are in close proximity to the endogeneous promoter of the target gene, it cannot be ruled out that under non-induced conditions a modulation of the target gene transcription may occur. Thus, the optimum amount of TetO and the optimal proximity between P_CMV_ and the endogeneous promoters (P_end_) may be determined empirically; however, this may be cumbersome and, timewise, costly. As the CRISPR/Cas9 technology allows for precise genome engineering, cell clones may be selected (assuming a mammalian cell carrying a diploid set of chromosomes) that carries the MAmTOW engineered cassette on only one allele, instead of two. An example of such a case is shown in Fig. 3c. One of the alleles carries the wild type transcription unit of the target gene, whereas the second allele is replaced by the regulatory cassette (it does not alter the target gene sequence or composition). In the latter, the endogenous promoter of the target gene is removed, thus this is subjected to regulation by only the introduced tetracycline-inducible cassette. Clones carrying mono-allelic replacements may be selected by cell-sorting when using a fluorescent reporter (Fig. 3c, lower panel). The selected mono-allelic clones exhibit wild type behavior when no tetracycline is added (albeit gene dosage may be halved), whereas addition of tetracycline leads to activation the second allele and gene (over)expression.

## Monitoring cell cycle robustness through systems biology

The proposed MAmTOW methodology represents a bridge between classic single-cell analysis techniques, such as flow cytometry, and modern techniques relying on quantitative (high-throughput) microscopy of CRISPR/Cas9-engineered cells. Directed gene replacement and gene manipulation can selectively alter protein dosage, leading to possible changes in spatial protein distribution; flow cytometry and microscopy can be used to monitor their effects on DNA content, allowing for protein visualization, thus providing information on timely molecular switches in cellular signaling. These switches are pivotal to control cell fate, such as the ones that govern cell cycle progression, where phase transitions are governed by modulation of the stoichiometry of activators and inhibitors that exploit their function within multi-protein complexes.

To understand how these transitions are regulated at a systems level by the balance between activator and inhibitor molecules, may reveal possible disease scenarios where protein dosage, kinetics and/or localization may be misregulated. That is, protein dosage, as well as localization are critical determinants of timely and ordered cell cycle transitions. However, an altered protein abundance or localization may impinge on the correct temporal dynamics, thus altering the capacity of the cell cycle to retain functionality against perturbations, thereby compromising cellular robustness. In both budding and fission yeasts it was shown that the upper limit of protein expression differs substantially between cell cycle proteins, in some cases without affecting cell viability.^48–50^ This result directly implies that cell cycle robustness against dosage is not identical for all molecules. Strikingly, the range of protein dosage allowed is not static, but it is dependent on the presence and, more specifically, the stoichiometry of the interactors. For some proteins, the dosage limit allowed can be increased by simultaneous overexpression of their interactor, suggesting that stoichiometry of protein complexes may be a crucial factor to maintain cell cycle robustness. Similar conclusions were drawn for another biological oscillator, the circadian clock. Specifically, in the mammalian circadian clock the stoichiometry between the CLOCK-BMAL1 (activator) and PER-CRY (inhibitor) protein complexes is crucial to maintain rhythm generation, thus the robustness of circadian rhythms.^60^ Thus, the proposed MAmTOW methodology may be generalized to also study circadian rhythms.

To investigate robustness of the cell cycle network at a systems level, integration of high-quality molecular interaction maps, which include localization of components, with sophisticated computer models is of vital importance. Computer models of cell cycle regulation can yield viable predictions on cell cycle robustness by varying dosage, which can be verified experimentally.^47, 61, 62^ Furthermore, recurring and new network motifs at the basis of system’s robustness may be identified.^63–67^ Therefore, models generated on the basis of molecular interaction maps may be simulated to mimic behavior of molecules and protein complexes at phase transitions, pinpointing to (i) the *precise* timing at which they function, and to (ii) the regulatory networks they establish with the surrounding molecules in different cellular compartments. Computationally, these models may include various exit codes that indicate whether and why an error in the previous iteration of the cell cycle simulation occurs.^48^ These exit codes can be used to determine whether the simulation algorithm should simulate progressive rounds of cell division, or if it should stop because the simulated cell has encountered a problem, e.g., incorrect ordering of temporal events, impossibility to trigger one of the checkpoints, numerical integration problems, etc. These error codes are related to the dynamics at a given moment that the model produces in terms of relative amplitudes and timing of the molecular species being simulated.

Modeling of cell cycle regulation has a long tradition, and kinetic models for the cell cycle in yeasts are particularly advanced.^40, 61, 68^ However, in this organism, alternative approaches such as qualitative modeling (also called Boolean models) have been employed to simulate cell cycle dynamics^67, 69–71^, also examining the timing robustness of the process with respect to checkpoint conditions.^72^ By comparison, fewer models exist that simulate the mammalian cell cycle^21, 73–76^, for which the availability of quantitative data about protein concentrations, localization and kinetics is still a challenge. To model in detail dynamics of the mammalian cell cycle control, reconstruction of a complete molecular interaction map shall incorporate theoretically information regarding localization, dosage, post-translational modifications, and complex formation. The complexity of this map as compared to the one of budding yeast^62^ may be reduced at the level of phase transitions, such as G1/S or G2/M, in order for both computer models and quantitative measurements to be manageable.^77^ By incorporating protein dosage as a function of time and localization, the temporal formation of protein complexes may be investigated with a detailed resolution. Again using the cyclin/Cdk inhibitor p27 as an example, we exemplify below the relevance of the interplay between dosage and timing in the context of cancer. In this scenario, the crucial tuning of p27 abundance and dynamics required for the correct progression throughout the successive cell cycle phases breaks down.

### p27 localization and dosage: timer to safeguard cellular proliferation

The temporal distribution of p27 is strictly regulated, exhibiting substantial fluctuation of p27 abundance and spatial dynamics over time. p27-mediated inhibition of Cdk2 prevents an untimely entry into S phase; contrarily, untimely p27-mediated Cdk-inhibition in S/G2 phases would lead to a failure of DNA synthesis, resulting in harmful, multiple rounds of DNA replication. For this reason, p27 is degraded timely, before the onset of cells into S phase, preventing unwanted Cdk2 inhibition.^78^ This evidence illustrates the need for a strict spatiotemporal regulation of p27 throughout cell cycle progression.

Early studies on p27 knockout mice showed that this Cdk inhibitor has a profound impact on organism and tissue development, with its absence leading to an increased body size and selective organ hyperplasia.^79–81^ The proliferative capacity of quiescent cells, such as neurons, appeared to be unaltered, whereas the proliferative capacity of immune cells was enhanced, proportionally to the body size, highlighting important cell and tissue-dependent specificity of p27 expression.^79^ The absence of p27 did not lead to an increase in spontaneous tumors; however, exposure of p27 nullizygous and heterozygous mice to gamma-irradiation or chemical carcinogens exhibited a predisposition to tumors in multiple tissues.^80^ p27 appears to safeguard cells against harmful insults, thus functioning as a tumor suppressor in mice. Furthermore, tumor suppressor activity of p27 was found to be dependent on its gene dosage.^80^ This role was further supported by investigating the phenotype of mice lacking Skp2, a component of an ubiquitin ligase that is part of the ubiquitin-proteasome pathway, which controls the fluctuations of p27 levels.^82^ Besides displaying a decrease in body weight due to increased p27 levels, these mice exhibit abnormal numbers of centrosomes; this phenomenon may be attributed to the presence of p27, as in Skp2^−/−^/p27^−/−^ mice centrosome abnormalities were much less pronounced.^83, 84^

p27 has been implicated as a prognostic factor for a number of tumors, as reduced p27 levels have been observed in cancers of the upper gastrointestinal tract, skin, glioma, sarcoma, and in haematopoietic and epithelial malignancies.^85^ However, a clear molecular mechanism is yet to be elucidated. Only recently the puzzle was partly solved, by showing that p27 serves as a safeguard tumor suppressor in malignancies where the axis pRB/p53, responsible for most anti-tumor mechanisms, is lacking.^86^ Although in these tumors p27 is unable to prevent S phase onset, it can inhibit cell division and, potentially, centrosome over-replication. This finding may explain the recurrent association of p27 with cancer severity, but also the ambiguity of p27 role in cancer as a function of dosage, localization and presence of other tumor suppressors. This evidence, together with the fact that a compromised compartmentalization, in particular an increase in its cytoplasmic localization^87^, can result in oncogenic functions of p27, indicates that this inhibitor may function as an intracellular timer at both G1/S and G2/M transitions as well as at cell division, where it modulates the centrosome number.^11, 12^ However, despite the substantial number of studies aiming to elucidate the canonical (Cdk-dependent) and non-canonical (Cdk-independent)^88, 89^ roles of p27, it still remains to be elucidated how its localization and dosage distribute through, and affect, cell cycle network dynamics.

**Fig. 4:**
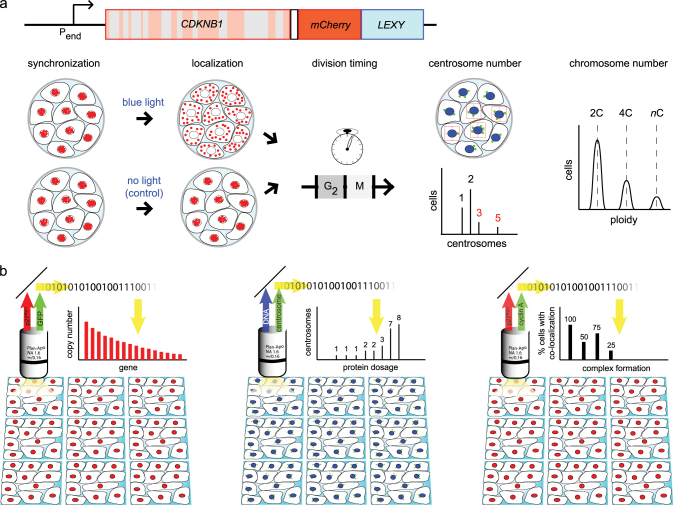
Light-inducible localization of p27 to study alteration of cell division. **a** The p27 endogenous locus is engineered to include a fluorescent reporter (mCherry) and a LEXY light-inducible domain (visualized in blue). A small spacer (white box) is placed between the *CDKNB1* gene coding for p27 and the reporter/LEXY fusion. mCherry is used instead of GFP, as the latter is not compatible with the blue light irradiation necessary to activate the LEXY domain. The engineered cells behave like wild type cells in the dark, but will delocalize p27 from the nucleus when irradiated with blue light. The effects of non-nuclear p27 on timing of the G2/M transition, centrosome number and ploidy may be evaluated. **b** Automated high-throughput fluorescence microscopic analysis may be used in single cells to investigate (from left to right): the upper expression limit of p27 (as well as of any cell cycle genes) by using the MAmTOW methodology; the impact of protein dosage and localization on the cell’s phenotype, such as the number of centrosomes; and the impact of protein dosage and localization for protein complex formation

As a dynamic regulator exhibiting fluctuation of absolute levels and localization throughout the cell cycle, p27 is an ideal candidate to explore cell cycle robustness. Therefore, we extend our ideas on the presented MAmTOW methodology and propose a strategy to dynamically modulate p27 localization by a light-inducible system, analyzing the phenotypic consequences. In this variant of the MAmTOW methodology, a fusion between the target gene *CDKNB1* coding for p27 and a fluorescent marker (in this specific case being mCherry) is extended to insert in its C-terminal region the aforementioned Light-inducible nuclear EXport sYstem (LEXY).^38^ Using LEXY, illumination by blue light enables the forced nuclear export of p27, thus allowing to investigate–through a changed localization–alteration of cellular properties such as timing at division, centrosome and chromosome numbers, with the latter reflecting the ploidy status of a cell (Fig. 4a). Strikingly, quantification of gene dosage may be realized to scale up the MAmTOW technology to high-throughput fluorescence microscopy (Fig. 4b), by which dosage (i) of a single target protein such as p27 (Fig. 4b, left panel), (ii) of a cellular property such as centrosome number (Fig. 4b, middle panel), and (iii) of two proteins forming protein complexes (Fig. 4b, right panel) may be quantified in single cells.

### Systems biology blueprint to uncover cell cycle functions in systems diseases

Cell cycle control is distributed over many components, and requires systems data to describe the role of its individual players. Therefore, data-driven (kinetic and stochastic) models of the biochemical networks that govern G1/S and G2/S transitions, as well as cell division have been developed to elucidate the cell cycle control structure. However, these models should allow for the understanding of how qualitative events (DNA replication initiation and mitosis) result from quantitative (de)regulation of cell cycle proteins in terms of their expression, subcellular localization and dosage. Because of the large number and complexity of the molecular interactions involved (feedback regulations, interplay between gene regulation, post-translational modifications, and localization), computer simulations of well-parameterized models are essential to design informative experiments. Such models should allow for a systematic exploration of how protein dosage and dosage imbalances within protein complexes impact phase transitions, thereby elucidating the relevance of protein dynamics for timing and duration of cell cycle switches. By incorporating realistic time and dosage constraints–which are lacking in current models of cell cycle regulation–it is possible to generate *precise* spatiotemporal computer models.

An example of successful modeling approach able to integrate quantitative information about gene dosage has been presented for the investigation of biochemical mechanisms driving the mammalian circadian clock.^90^ A computer model was proposed to rationalize the early finding that stoichiometry between activators and repressors, rather than their absolute levels, is crucial to maintain a robust circadian rhythm.^60^ Specifically, the model is able to show that the biochemical mechanism underlying oscillations of the circadian clock relies on the stoichiometric balance between activators and inhibitors. By varying the ratio between circadian clock components, differences in circadian rhythms between cell types were explained, and effects of individual clock factor expression on circadian robustness were predicted.

Putative interactions or protein co-dependencies may be investigated by model simulations with altered dosage and/or localization of two proteins simultaneously vs. model simulation of the same proteins independently.^47^ The compartmentalization aspect would yield hypotheses testable experimentally on the quantitative importance of stoichiometry in protein complexes and localization on the *precise* timing of cell cycle transitions, and how these impact on the overall system robustness. These properties cannot be captured in current cell cycle models, as they currently do not include information about spatiotemporal dynamics. The approach that we have proposed can result in an increased model accuracy. Specifically, the modified models may be interrogated for the effects (i) of simultaneous dosage variation of partners within a protein complex, and (ii) on the phase transition timing, which impact on cell cycle robustness. Strikingly, in disease scenarios, these models may predict drug treatment more accurately as compared to pure statistical models (for example, it was suggested that pRB/p53 null tumors should not be treated with inhibitors of DNA synthesis, but with Skp2 inhibitors, to increase p27 levels^86^). Model predictions may subsequently be tested by modifying mammalian cell lines to express two proteins with a mutual dosage dependence, and comparing the dosage threshold with cell lines containing only one single protein, akin to the approach used in the yeast gTOW method to investigate protein complex stoichiometry. If our proposed transient (plasmid) system is used, co-expression of the two proteins is realized by using episomes with different fluorescent reporters. When varying the ratio of plasmid transfected, availability of either protein for complex formation will be altered; by using two fluorescent reporters as readouts, information on the boundaries of protein complex stoichiometry may be retrieved, as determined by protein availability.

Thus, a systematic exploration of model predictions by using quantitative data obtained employing the MAmTOW methodology and localization studies, may result in focused disease treatment strategies based on the understanding of the underlying process rather than on correlative deductions.

**Fig. 5:**
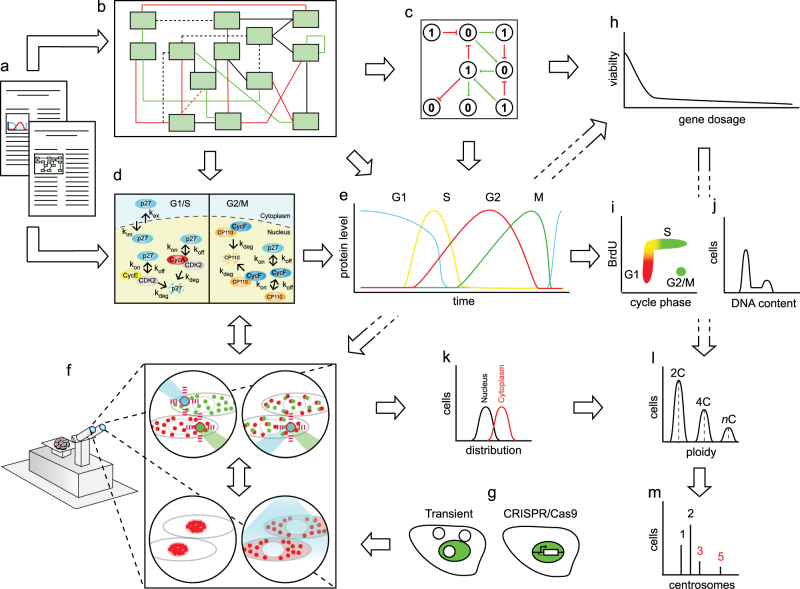
Systems Biology blueprint to uncover new cell cycle functions. A framework that integrates computational and experimental analyses is proposed to elucidate the impact of protein dosage, localization, and complex formation on temporal dynamics of cell cycle progression. Literature analysis **a** allows for mapping of all molecules involved in the cell cycle network **b**, and assigning the directionality of their connections. This information serves to generate cell cycle models **c** by employing alternative computational strategies (e.g., kinetic, stochastic, Boolean) which rely on the spatiotemporal information for each molecule **d**. These models describe the behavior of molecules over time **e** and will provide testable hypothesis, with a particular focus on how protein spatiotemporal dynamics may impact on cell cycle robustness, progression and control. We expect these properties to emerge from the non-linear ensemble of molecules. The generated hypotheses will be tested by employing a number of advanced expertimental technologies, and challenge them to: (i) investigate protein relocalization and protein complex formation by light-induced technology and Fluorescence Correlation Spectroscopy, respectively **f**, and (ii) investigate gene dosage by transient and stable (CRISPR/Cas9-based) MAmTOW methodology **g**. Furthermore, a variety of readouts may be employed to measure gene dosage **h**, cell cycle phase distribution by FUCCI system **i**, DNA content **j** cytoplasmic and nuclear protein distributions **k**, cellular ploidy **l**, and centrosome number **m**

The Systems Biology strategy that we propose therefore integrates computational and experimental challenges to investigate how protein dosage and localization of cell cycle molecules affect temporal dynamics and robustness at the systems level. This challenge may be realized practically by integrating well-established computational frameworks (such as kinetic, stochastic and/or Boolean models) and molecular map reconstruction with the development and optimization of experimental efforts (Fig. 5). Computer models, predicting how perturbed protein dosage and localization impinge on cell cycle robustness, may serve as an input for hypotheses, testable experimentally, of the extent by which cell cycle robustness is maintained. Experimental testing would not just be of support to the models, but is meant to determine the endpoints of cell cycle perturbations; do these lead to cell cycle arrest, apoptosis, or to potential pathological conditions such as aneuploidy or abscission defects? Thus, the blueprint (Fig. 5) requires integration of i) state-of-art molecular tools, such as the CRISPR/Cas9 technology, that allow precise gene replacements in mammalian cells, (ii) emerging, practically realizable technologies to control gene expression in real-time, such as the innovative MAmTOW methodology that we propose here, and (iii) computational modeling and network analysis of a curated molecular map, to compute robustness and determine responsiveness quantitatively. This is pivotal to assess whether and how alteration of the complexity of the cell cycle network, inspected by removing interactions in silico, impacts on its robustness against perturbations.

## Outlook

A key, yet elusive question in biology is: ‘Why are cellular networks so complex?’ A possible answer may be that complexity is required to lend cellular processes flexibility to respond timely to a variety of dynamic signals, while simultaneously warranting robustness to protect cellular integrity against perturbations. One example of such a biological process is the cell cycle. Some mammalian cell types retain the ability to divide and, importantly, do so continuously, whereas others require specific inputs to re-enter the cell cycle. For example, upon terminal differentiation, neurons typically halt cell division indefinitely; however, this is not due to a loss of the capability of cell division as, when stimulated by the right cues, neurons can initiate the cell cycle, even when undesired.^91^ Conversely, T-cells and hepatocytes are quiescent until reception of immunological signals and after liver damage, respectively, after which they promptly enter the cell cycle.^92, 93^ Yet other cell types, such as stem cells, important to the development of the epidermis, cells of the hematopoietic system and the intestine, retain somatic replicative capacity whereas their differentiated counterparts loose this functionality.^94^ Importantly, by supplying the known ‘Yamanaka factors’, even differentiated cells can re-acquire pluripotent properties and, concomitantly, increase proliferative capacity.^95^ Not only the cycling program, but also the stimuli required to induce cell cycle entry and proliferation, differ greatly among the aforementioned cell types, which leads us to hypothesize a plasticity of cell cycle control.

Despite this impressive flexibility in induction, the cell cycle appears rather ‘rigid’, being tightly regulated: untimely stimuli for proliferation may lead to halt cell division and, eventually, to apoptosis. The existence of several molecular safeguards that act upon reception of external stimuli guarantees halting; among these safeguards, p27 is a pivotal player. Strikingly, in all of the aforementioned cell types, e.g., neurons, T-cells, hepatocytes and stem cells, this cyclin/Cdk inhibitor plays crucial roles.^96–99^ This evidence suggests that dosage and spatiotemporal regulation of this cell cycle regulator, among others, may critically determine developmental states and cellular fates, such as pluripotency and differentiation. Interestingly, a computer model that investigates the effect of gene dosage for a robust circadian clock^90^ also elaborates on the link between components of the clock and the tumor-suppressor p53. Specifically, the computer model was able to capture the observed experimental oscillations of Per2 and p53 only when protein localization was incorporated. As a number of cell cycle components are regulated by p53, Per2 may link circadian clock and cell cycle.^5^ In this context, regulation of p53-mediated p27 stability through activation of the ubiquitin ligases Pirh2 and KPC1 may provide a connection between the two networks. Considering that deregulation of p53 as well as modulation of p27 levels are implicated in cancer progression^86^, it would be worthwhile to investigate this possible biochemical mechanism by dedicated computer models that integrate dosage constraints and spatiotemporal information.

Thus, the Systems Biology blueprint that we present here may yield and improve diagnostic predictions, and forecast loss of robustness in pathological disorders.

## Electronic supplementary material

Supplementary Information

Supplementary Figures
