# Supplementary material for: Quantitative Systems Biology to decipher design principles of a dynamic cell cycle network: the “Maximum Allowable mammalian Trade–Off–Weight” (MAmTOW)
Source: NPJ Syst Biol Appl. 2017 Sep 19;3:26. doi: 10.1038/s41540-017-0028-x (PMC5605530; doi:10.1038/s41540-017-0028-x)
Supplement: Supplementary file 1 — Supplementary Information [file 41540_2017_28_MOESM1_ESM.docx]

**Supplementary Information**

**Quantitative systems biology to decipher design principles of a dynamic cell cycle network: the “Maximum Allowable mammalian Trade–Off–Weight" (MAmTOW)**

Matteo Barberis, Paul Verbruggen

**Supplementary References (see Figure 1 Legend)**

1. Starostina, N. G. & Kipreos, E. T. Multiple degradation pathways regulate versatile CIP/KIP CDK inhibitors. *Trends Cell Biol.* **22**, 33–41 (2012).
2. Yoon, M.-K., Mitrea, D. M., Ou, L. & Kriwacki, R. W. Cell cycle regulation by the intrinsically disordered proteins p21 and p27. *Biochem. Soc. Trans.* **40**, 981–988 (2012).
3. Susaki, E., Nakayama, K. & Nakayama, K. I. Cyclin D2 translocates p27 out of the nucleus and promotes its degradation at the G0-G1 transition. *Mol. Cell. Biol.* **27**, 4626–4640 (2007).
4. Connor, M. *et al.* CRM1/Ran-mediated nuclear export of p27Kip1 involves a nuclear export signal and links p27 export and proteolysis. *Mol. Biol. Cell* **14**, 201–213 (2003).
5. Rodier, G. *et al.* p27 cytoplasmic localization is regulated by phosphorylation on Ser10 and is not a prerequisite for its proteolysis. *EMBO J.* **20**, 6672–6682 (2001).
6. Boehm, M. *et al.* A growth factor-dependent nuclear kinase phosphorylates p27Kip1 and regulates cell cycle progression. *EMBO J.* **21**, 3390–3401 (2002).
7. Fujita, N., Sato, S., Katayama, K. & Tsuruo, T. Akt-dependent phosphorylation of p27Kip1 promotes binding to 14-3-3 and cytoplasmic localization. *J. Biol. Chem.* **277**, 28706–28713 (2002).
8. Kamura, T. *et al.* Cytoplasmic ubiquitin ligase KPC regulates proteolysis of p27(Kip1) at G1 phase. *Nat. Cell Biol.* **6**, 1229–1235 (2004).
9. Viglietto, G. *et al.* Cytoplasmic relocalization and inhibition of the cyclin-dependent kinase inhibitor p27Kip1 by PKB/Akt-mediated phosphorylation in breast cancer. *Nat. Med.* **8**, 1136–1144 (2002).
10. Shin, I. *et al.* PKB/Akt mediates cell-cycle progression by phosphorylation of p27 at threonine 157 and modulation of its cellular localization. *Nat. Med.* **8**, 1145–1152 (2002).
11. Liang, J. *et al.* PKB/Akt phosphorylates p27, impairs nuclear import of p27 and opposes p27-mediated G1 arrest. *Nat. Med.* **8**, 1153–1160 (2002).
12. Kossatz, U. *et al.* Skp2-dependent degradation of p27kip1 is essential for cell cycle progression. *Genes Dev.* **18**, 2602–2607 (2004).
13. Malek, N. *et al.* A mouse knock-in model exposes sequential proteolytic pathways that regulate p27Kip1 in G1 and S phase. *Nature* **413**, 323–327 (2001).
14. Besson, A. *et al.* A pathway in quiescent cells that controls p27Kip1 stability, subcellular localization, and tumor suppression. *Genes Dev.* **20**, 47–64 (2006).
15. Lu, Z. & Hunter, T. Ubiquitylation and proteasomal degradation of the p21(Cip1), p27(Kip1) and p57(Kip2) CDK inhibitors. *Cell Cycle* **9**, 2342–2352 (2010).

**Supplementary Figure Legends**

**Figure S1.** Fluorescent Ubiquitination-based Cell Cycle Indicator (FUCCI) to visualize protein localization through time. Cdt1-RFP accumulates in the nucleus during G1 phase, whereas Geminin-GFP is degraded. Thus, nuclei revealing red fluorescence belong to cells in G1 phase. In S phase, both Cdt1-RFP and Geminin-GFP are present, yielding a blend of the red and green colours. As cells progresses through S phase and enter G2 phase, Cdt1-RFP is degraded whereas Geminin-GFP’s signal dominates. Thus, green colour indicates cells in G2 phase. During mitosis and cytokinesis, both Cdt1-RPF and Geminin-GFP are degraded, leaving cells devoid of any fluorescent signal.

**Figure S2.** Light-inducible systems to alter, reversibly, spatiotemporal dynamics. (**a**) Light-Inducible Nuclear localization Signal (LINuS). The prototype of a transcription unit encoding the mCherry/LINuS protein fusion is represented. Gene target exons are shown in red colour, whereas introns in grey colour. A small glycine-serine stretch is depicted in white colour followed by a downstream exon encoding both mCherry and LINuS elements. A cytoplasmic protein is directed to the nucleus after blue light exposure; the effect is promptly reversed if the light is switched off. The diffusion of the protein can be followed in time by visualization and imaging of the red mCherry fluorescent reporter. (**b**) Light-inducible nuclear EXport sYstem (LEXY). The prototype of a transcription unit encoding the mCherry/LEXY protein fusion is represented. Colours indicating the gene target as well as the exon encoding mCherry and LEXY elements are shown as in (**a**). Contrarily to the LINuS sequence in (**a**), the LEXY sequence enables targeting of a protein from the nucleus to the cytoplasm; the effect is promptly reversed if the light is switched off. (**c**) Light-inducible protein translocation in single-cells. Targeting individual cells by low-power laser irradiation enables single-cell protein translocation studies as compared to laser irradiation of a cell population, as shown in (**a**) and (**b**). Prolonged irradiation of the nucleus leads to protein translocation to the cytoplasm; the effect is promptly reversed if the light is switched off.

**Figure S3.** Protein Quantitation Ratioing (PQR) to quantify protein dosage. PQR ensures equimolar fluorescent reporter and target protein production, resulting in protein ratiometric readouts. The prototype of an engineered transcription unit encoding the PQR sequence (in blue colour) and a fluorescent reporter (in green colour) is shown. Gene target exons are shown in red colour, whereas introns in grey colour. Transcription of the unit leads to the production of an mRNA that is translated to a polyprotein consisting of the fluorescent reporter fused to the PQR sequence and the target gene at the N-terminus. The PQR element leads to separation of the two polypeptides by interfering with the translation by the ribosome, thus effectively leading to its removal (process shown by the scissors between the fluorescent reporter and the target gene). After removal of the PQR peptide, fluorescent reporter and target protein are separated and present in a 1:1 ratio in their native conformations.

**Figure S4.** Fluorescence microscopy to quantify protein dosage. (**a**) Fluorescence Correlation Spectroscopy (FCS). A small volume of a cell either in the nucleus or in the cytoplasm (cell interior) is shown by a framed interior. The cell interior is illuminated by a laser through a pinhole generating a light cone (in blue colour) through the cell. The green circles indicate moving fluorescently-labelled particles, whereas the dark blue area in the middle of the light cone indicates the confocal volume (labelled with “c”). A fluorescent particle, for instance a protein fused to GFP, fluoresces brightly when diffusing through the confocal volume. The intensity of this signal is measured and registered in time. The duration of the fluorescent pulse carries information on the diffusion speed of the particle, while the frequency of pulses carries information on the particle’s concentration. By determining the amount of fluorescence events with a specific τ (arbitrary time interval) after each time t, an autocorrelation graph (G_AC_(τ)) can be obtained. From this graph information on protein concentration and diffusion are retrieved (for an extensive review, see ref. 55). (**b**) Fluorescence Cross-Correlation Spectroscopy (FCCS). If two different fluorophores are analysed, the correlation between the fluorescence graphs can be used to investigate protein-protein interactions and complex formations. If no interaction occurs between a green-fluorescent particle and a red-fluorescent particle, these will traverse the confocal volume independently, resulting in a no correlation between the two fluorescent signals. Conversely, if red and green fluorescent particles interact, their signals will overlap.
