# Supplementary figures and images for: Quantitative Systems Biology to decipher design principles of a dynamic cell cycle network: the “Maximum Allowable mammalian Trade–Off–Weight” (MAmTOW)
Source: NPJ Syst Biol Appl. 2017 Sep 19;3:26. doi: 10.1038/s41540-017-0028-x (PMC5605530; doi:10.1038/s41540-017-0028-x)

Figure S1

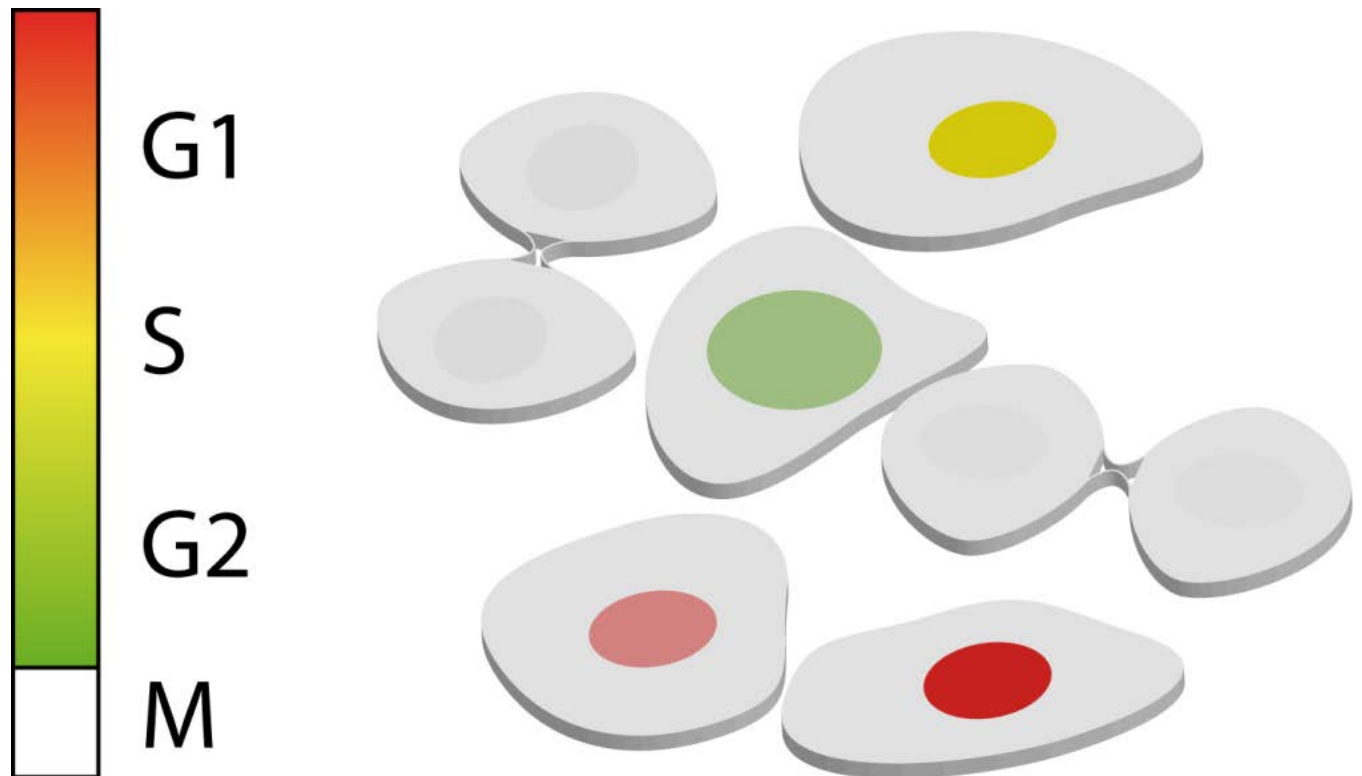

Figure S2

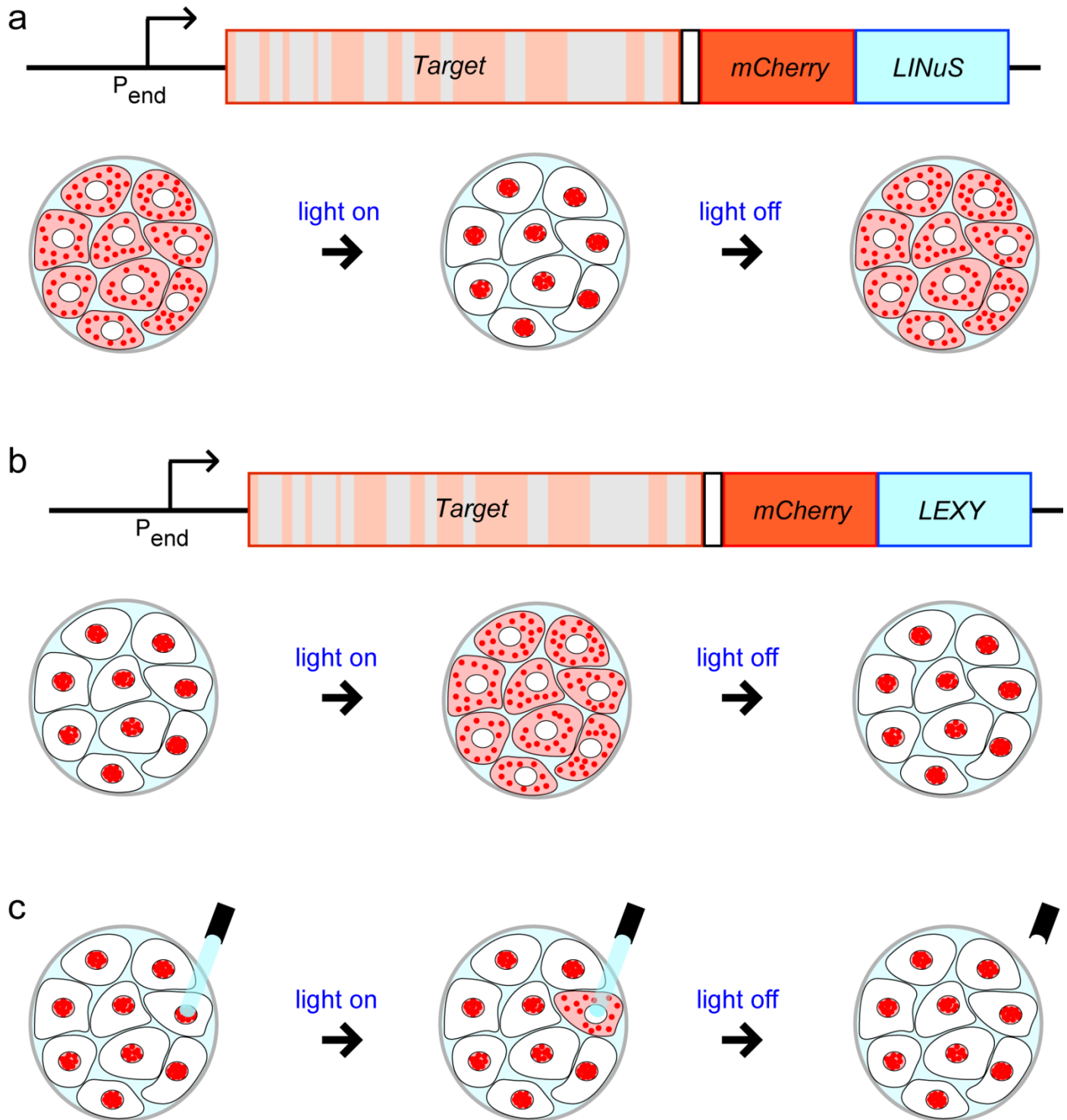

Figure S3

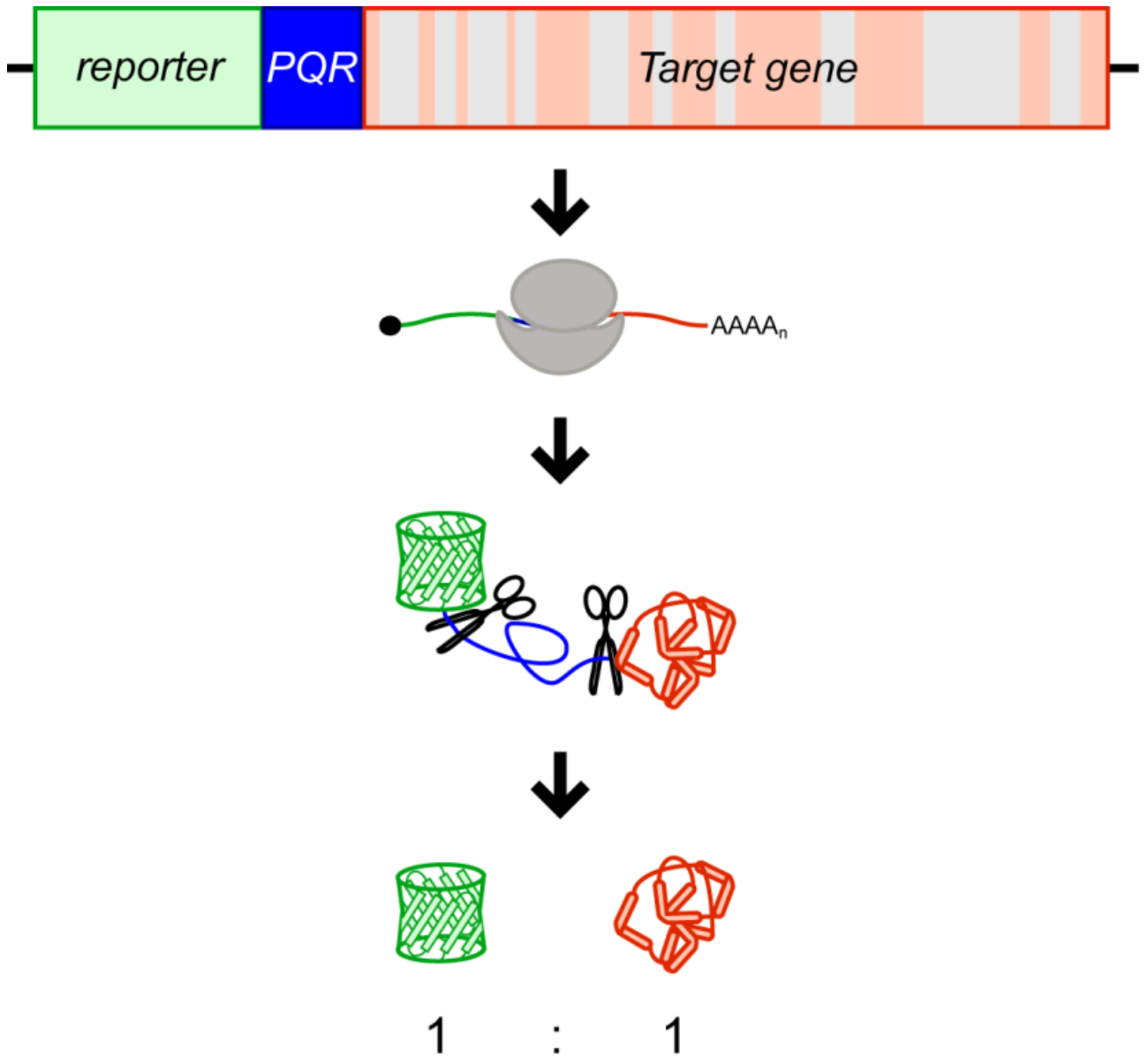

Figure S4

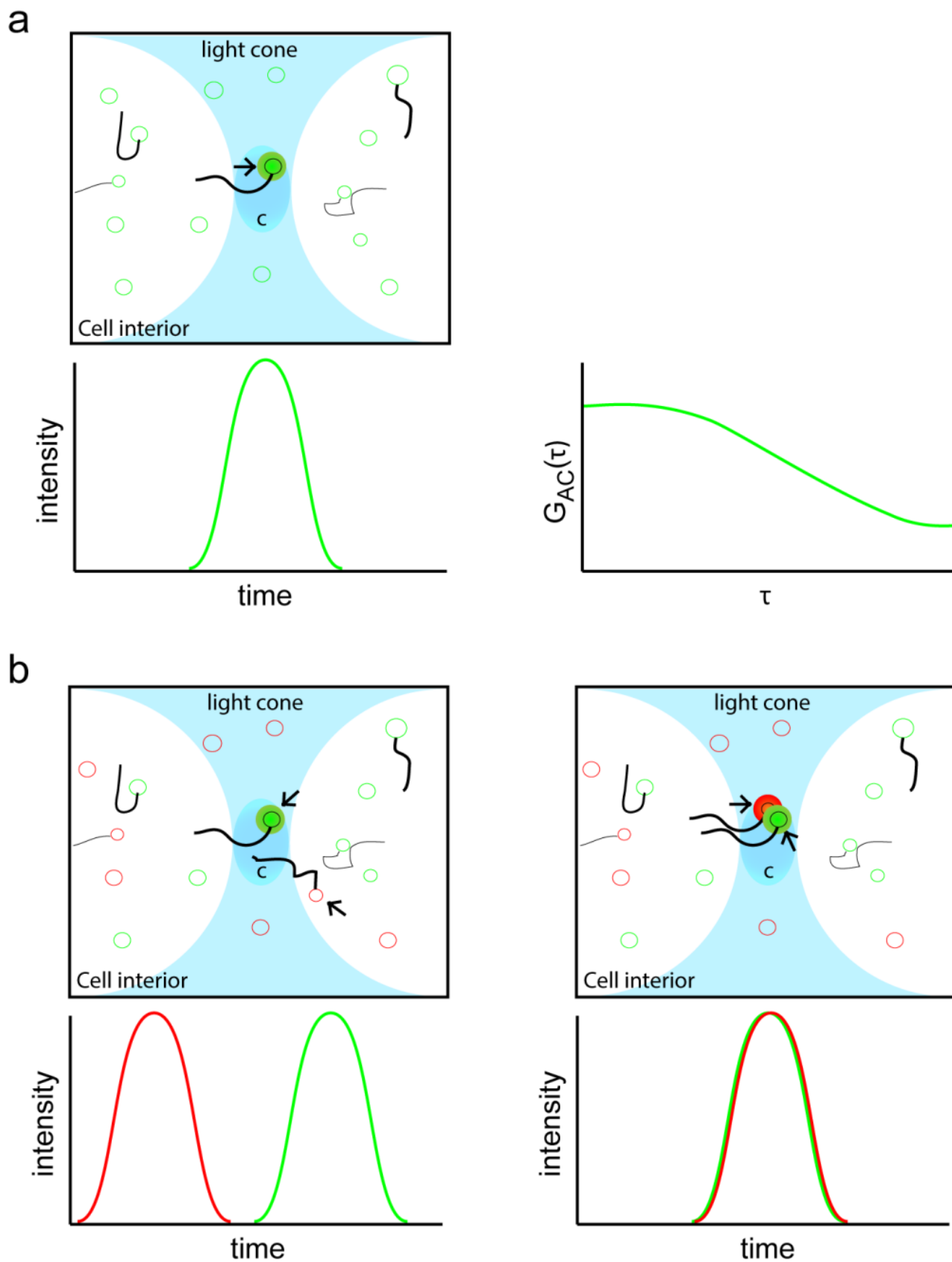

Supplement: Supplementary file 2 — Supplementary Figures [file 41540_2017_28_MOESM2_ESM.pdf]
